# Faith Cures

**Published:** 1883-01

**Authors:** George E. Blackham


### FAITH CURES.

GEO. E. BLACKHAM, M. D., F. R. M. S.

That mental, moral and emotional causes may affect the physical conditions of the body is well known. SHAME dilates the capillary blood-vessels of the face and the increased blood supply is shown by the rosy hue or "blush" as it is called. FEAR, on the other hand, contracts them and gives the face a pallid hue. ANGER, like shame, dilates the capillaries of the face, and the effects of sudden rage are well described by Scott, in his poem of Marmion:

> "On the Earl's cheek the flush of rage,
> O'ercame the ashen hue of age."

These results are produced by disturbances of the supply of nervous force sent from the nerve centers to the vaso-motor nerves—but the effect of these emotional disturbances of the great nerve centers is by no means confined to the minuter blood-vessels of the skin of the face—the heart itself is often affected. The phrases, "my heart jumped for joy," "my heart stood still with fear," are but popular and extravagant expressions of widely observed facts, showing the powerful influence of the emotions upon the circulatory system through the medium of the nerves. The muscles may be profoundly affected. Every physician, and many laymen can recall instances of convulsions superinduced in highly susceptible, nervous subjects, by sudden emotions of fear or joy. Sensibility may be heightened to a painful extent. In moments of anxious suspense, sensibility is often increased to a wonderful degree, the special senses are specially influenced. "It seemed as though I could hear a pin drop," is the common expression of this condition. On the other hand, mental exaltation often produces physical insensibility. Soldiers have been gravely wounded and, in the excitement of a charge, have remained unconscious of it till loss of blood has produced physical exhaustion. Martyrs have gone smiling to the stake and borne the pangs of fiery death with apparent unconsciousness of suffering. The vital functions are interfered with in many ways by emotional disturbances or impressions.

Shakespeare, with his usual insight, gives many instances. Macbeth bids the guests welcome to his coronation feast, and says:

> "Now good digestion wait on appetite,
> And health on both."

And is ready to join in the feasting till he sees the ghost of the murdered Banquo rise before him, when appetite and digestion both disappear under the influence of the emotion of horror which takes possession of him.

Dr. Carpenter remarks, "that the secretion of gastric juice is affected in a very marked manner by conditions of the nervous system, is indicated by the effect of the mental emotions, in putting an immediate stop to the digestive process, when it is going on at full vigor." And Dr. Milner Fothergill, quoting this with approval, adds: "We are all only too familiar with the consequences of bad news, or other upset while at meals."*

Dr. Carpenter again says: "The flow of *saliva*, again, is stimulated by the sight, the smell, the taste, or even by the *thought* of food; especially such as is of a savory character. On the other hand, violent emotion may suspend the salivary secretion, as is shown by the well known test, often resorted to in India, for the discovery of a thief among the servants of a family, that of compelling all the parties to hold a certain quantity of rice in the mouth during a few minutes, the offender being generally distinguished by the comparative dryness of his mouthful at the end of the experiment. There is much reason to believe that the secretion of the gastric fluid is affected in the same manner as that of the saliva. * * * That the secretion is entirely suspended by powerful emotion, seems almost certain, from the well known influence which this has in dissipating the appetite for food, and in suspending the digestive process when in active operation. As a cheerful state of feeling, on the other hand, seems to be decidedly favorable to the performance of the digestive functions, it probably exerts a beneficial influence as to both quantity and quality, in the secretion of gastric fluid."

Again he says: "The influence of the state of *expectant attention* in modifying the processes of nutrition and secretion, is not less remarkable than we have already seen it to be in the production or modification of muscular movements. It seems certain that the simple *direction of the consciousness* to a part, independently of emotional *excitement*, but with the *expectation* that some change will take place in its organic activity, is often sufficient to induce such an alteration, and would probably always do so if the concentration of the attention were sufficient. * * * In many individuals especially females) whose sympathies are strong, a pain in any part of the body may be produced by witnessing it in another, or even hearing described the sufferings occasioned by disease or injury of that part; and, if this pain be attended to and believed in, as an indication of serious mischief, injurious consequences are very likely to follow. So, again, the self-tormenting hypochondriac will imagine himself the victim of any malady he may 'fancy,' and, if this fancy should be sufficiently persistent and engrossing, it is not unlikely to lead to real disease of the organ to which it relates."

*Fothergill on Indigestion and Billiousness.

It is evident, then, that the emotions pain, pleasure, hope, fear, and imagination, may influence the vital processes, that these processes are specially liable to be influenced by expectant attention directed towards them; but thus far we have only looked upon the darker side of the picture, the evil effects of these mental conditions; but every power for good is a power for evil if misdirected, and *vice versa*. Emotion may produce disease, *expectant* attention directed to some special organ may actually produce in it the looked for disease; but, on the other hand, emotion may be the cause of a return to health. The love-sick maiden is very apt to improve in health when the course of true love begins to run smooth. *Expectant attention* directed to a diseased organ with the belief in its restoration to health may be a potent factor in that restoration.

If you can get the hypochondriac once *convinced* that he is on the mend, his recovery will be rapid. Herein lies the secret of many of the so-called "faith cures" which sensation-loving reporters expatiate upon from time to time. The subjects are mostly, as might be expected, hysterical women whose maladies, like their cures, are mainly creations of the patient's imagination. The cures are often marvellous, and are believed by patients and friends to be miraculous.

Indeed, to question their miraculous nature is often looked upon by worthy and by no means weak-minded people as flat blasphemy —but yet there is in them no element of the supernatural. The troubles amenable to the "faith cure" are chiefly, if not exclusively, *functional* disorders of nervous origin. Paralytics have been made to walk, bed-ridden spinsters to rise and resume the duties of life, neuralgias have vanished and dyspepsias disappeared, nervous blindness has been cured, but broken bones have not united on the instant, cataracts have not become transparent, cancers have not been healed nor have any profound *organic* lesions been relieved. No faith has ever, in modern times at least, restored the amputated arm or the maimed foot. Imaginary maladies have yielded to imagination, that is all. And even in allowing this much, the student of neuropathology feels as though he must guard himself from unintentional misrepresentation. Not all cases of apparent cure by "faith" are genuine cures of even functional troubles. A large proportion of them are pure fraud, from beginning to end. Fraudulent disease as well as fraudulent cure. Women in perfect physical health have often taken to their beds, moved thereto by a morbid egoism, a desire for sympathy, attention, consideration, a wish to be the center of talk, of wonder, of mystery, and their apparent cure has been the result of similar notions, coupled often with a weariness of the role of invalid, which had been carried on till the novelty has faded out and the assiduous attentions of sympathising friends have been exhausted. The cure by "faith" tickles this morbid egoism with the notion that for *them* a special interposition of the Almighty is to be made, that *they* are of sufficient importance for Him to set aside in their behalf the laws which he has established "from the beginning" for ordinary mortals, and by the hope that their recovery, like their illness, may be "a nine days' wonder" and attract to them that attention which their natures crave.

It is useless to argue with those who really believe in "faith cures" as direct interpositions of the power of the Almighty. Useless to tell them that He is "without change or shadow of turning," that the laws he established in the beginning have never been broken or set aside.

Faith is an important element of cure in all cases of illness, every physician knows that, and every sensible physician tries to secure the faith of his patients, but the action of faith, whether as the sole means of cure or as an important adjunct to rational and physical means, is purely physiological and never supernatural.

Send in your subscription for the coming year.

